# Leukocytoclastic Vasculitis following the First Dose of the Elasomeran COVID-19 Vaccination

**DOI:** 10.1155/2022/1469410

**Published:** 2022-08-04

**Authors:** Jarett J. Casale, Mikél E. Muse, Tara J. Snow, Karen P. Gould, Natalie D. Depcik-Smith

**Affiliations:** Campbell University at Sampson Regional Medical Center, Clinton 28328, NC, USA

## Abstract

Our case highlights leukocytoclastic vasculitis as a potential side effect of the elasomeran COVID-19 vaccine. As the elasomeran vaccine becomes more widely available to the public, cutaneous reactions should be noted and looked for as potential side effects of the vaccine. Our patient had a history of immune thrombocytopenic purpura, making this a potential predisposing condition to the development of vasculitis following elasomeran administration. The case of vasculitis in our patient, although diffuse in distribution, was self-resolving. Our patient was counseled of the potential risk of worsening reaction to the second dose of the vaccine and instructed to proceed at their own risk. He elected to receive the second vaccination dose without any further reaction or side effects. Primary teaching points from this case include the potential of developing leukocytoclastic vasculitis following the elasomeran vaccination. Patients who develop LCV following the first dose should be counseled of the risks associated with receiving the second dose, including progression to systemic organ involvement.

## 1. Introduction

Leukocytoclastic vasculitis refers to inflammation of small blood vessels resulting from immune-complex deposition within blood vessel walls leading to tissue destruction [1]. Infections and medications are the most common identifiable causes of leukocytoclastic vasculitis; however, up to 50% of cases are idiopathic in etiology [2]. The clinical course is often self-limiting and resolves with avoidance of the offending agent [1, 3]. Treatment is primarily supportive but may include systemic corticosteroids or other more potent immunosuppressive agents for severe cutaneous disease [2, 3].

We present a patient with leukocytoclastic vasculitis that developed 72 hours after administration of the first dose of the Spikevax (elasomeran) vaccination developed by Moderna. This vaccination is a two-part vaccination that utilizes mRNA technology to promote an immunological response to the spike protein of the Sars-CoV-2 virus. While the efficacy and safety of mRNA vaccination technology have been studied at length, the COVID-19 pandemic is the first time in which widespread utilization of this vaccine technology has been employed. This case highlights leukocytoclastic vasculitis as a potential rare side effect of the COVID-19 vaccination.

## 2. Case Synopsis

**Figure 1:**
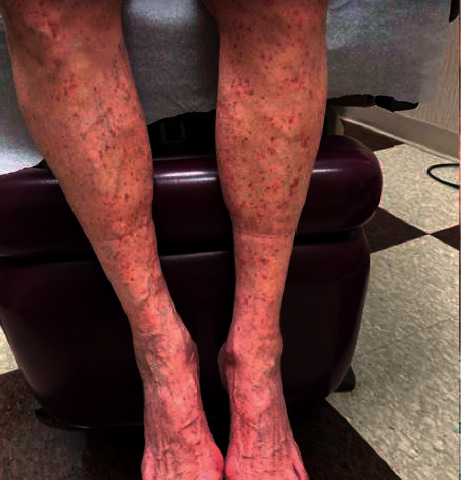
Image of the patient's bilateral lower legs demonstrating symmetrically distributed small, red, violaceous petechiae.

**Figure 2:**
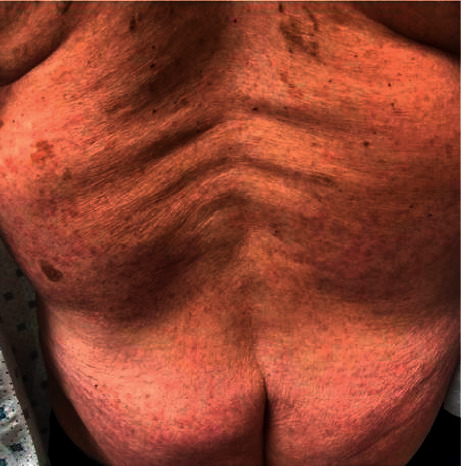
Image of the patient's back demonstrating symmetrically distributed small, red, violaceous petechiae.

An 80-year-old male with a history of immune thrombocytopenic purpura (ITP) presented for evaluation of multiple small, red, violaceous petechiae involving the bilateral dorsal feet, legs, thighs, buttocks, and arms (Figures 1and 2) that appeared 72 hours after his first dose of the vaccine. The patient denied any recent illness, fever, weight changes, weakness, fatigue, shortness of breath, chest pain, myalgias, arthralgias, abdominal pain, chest pain, dyspnea, cough, neuropathy, or changes to his medications. Current medications include atorvastatin taken for 12 years, alprazolam as needed for 3 years with no recent increase in use, losartan taken for 8 years, carvedilol taken for 3 years, aspirin taken for 12 years, and fluticasone as needed for 5 years with no recent increase in use. A 4 mm punch biopsy was taken from the right thigh.

**Figure 3:**
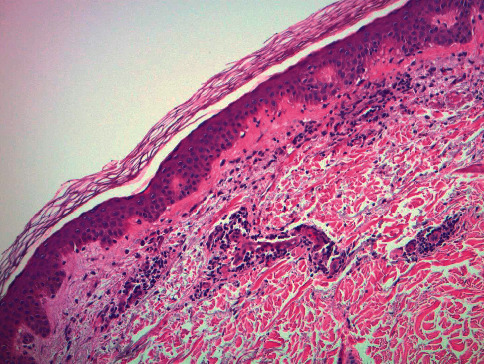
H&E (10x) shows a perivascular infiltrate with fibrinoid changes consistent with leukocytoclastic vasculitis.

**Figure 4:**
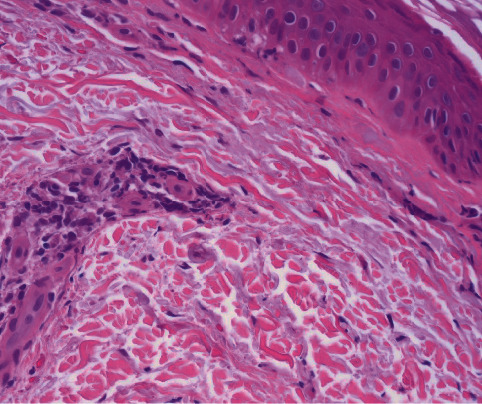
H&E (40x) shows early fibrinoid changes and scattered neutrophils consistent with leukocytoclastic vasculitis.

Histology showed a perivascular lymphocytic infiltrate with extravasated red blood cells suggestive of vascular injury (Figures 3 and 4). Fibrinoid changes of the lumina and red cell extravasation without the involvement of deep vessels were observed. Pathology favored an early evolving leukocytoclastic vasculitis secondary to a vaccine reaction given the clinical history of the patient.

The patient was managed supportively and seen for follow-up 10 days after the initial biopsy was taken. Physical examination at that time revealed nearly complete resolution of the initial lesions. The patient was counseled of the potential risks associated with receiving the second vaccine dose including worsening cutaneous reaction or potential for more severe systemic involvement and instructed to proceed at their own risk. The patient elected to receive the second dose of the elasomeran and denied any skin reactions.

## 3. Case Discussion

Leukocytoclastic vasculitis (LCV) is caused by the deposition of immune complexes within blood vessels leading to immune system activation and fibrinoid necrosis, resulting in characteristic vasculitic skin lesions [1]. The etiology of LCV is 45–55% idiopathic, 15–20% infectious, 15–20% autoimmune connective tissue disease or inflammatory disorders, 10–15% drug-induced hypersensitivity reactions, and 5% malignancy or lymphoproliferative disorders [2]. Although exceedingly rare, LCV has been reported as a side effect of the influenza vaccine [4]. The SARS-CoV-2 (COVID-19) pandemic has resulted in the United States Food and Drug Administration Emergency Use Authorization of two highly effective vaccinations from Moderna and Pfizer-BioNTech using mRNA vaccination technology [5].

Messenger RNA vaccine technology has been studied at length and offers strong safety advantages compared to traditional vaccine technology [6]. The primary advantage is due to the vaccine containing only genetic material needed for the expression of a selected encoded protein, rather than direct inoculation with a given antigen. In addition to mRNA, both the Moderna and Pfizer-BioNTech vaccines contain various other preservative compounds including the preservative propylene glycol. The elasomeran vaccine also contains the lipid compounds SM-102 (proprietary to Moderna), 1,2-distearoyl-sn-glycero-2-phosphocholine, cholesterol, inactive salts, and buffers such as tromethamine hydrochloride, acetic acid, and sodium acetate. We are uncertain which vaccine ingredient was the likely immune reactant leading to the development of LCV in our patient from the first dose. Previous studies of cutaneous reactions due to the elasomeran vaccine have found that the incidence of a delayed reaction after the first dose is more common on days 7 and 8, while a reaction with the 2nd dose is likely to be less severe and develop faster within 1–3 days, without severe adverse or allergic events [7]. Our patient did not report any symptoms following the second dose of the vaccine.

LCV presents clinically as symmetrically distributed palpable purpura or infiltrated erythema most commonly on the bilateral lower extremities [2, 3]. Deposition of immune complexes in LCV is predominantly limited to superficial postcapillary venules, leading to complement activation and neutrophil recruitment [2]. This results in fibrinoid necrosis of vessel walls, leading to edema of endothelial cells and red blood cell extravasation [1, 2]. Extravasation of red blood cells causes the characteristic macroscopic findings of inflammatory purpura, which can be further examined by diascopy. The lesions will often demonstrate a blanchable component due to underlying inflammation, and a nonblanchable component due to subcutaneous hemorrhage [2].

LCV is diagnosed by punch biopsy preferably within 18–48 hours of lesion onset. After this time frame, dissipation of immune complexes and recruitment of various mononuclear cells in addition to neutrophils makes the histopathological findings less specific for LCV [2]. In addition to H&E, a biopsy for direct immunofluorescence is often helpful to narrow the differential diagnosis by distinguishing among various immunoglobulin causes, such as IgA-associated Henoch–Schönlein purpura [3]. In cases of LCV with evidence of systemic involvement based on a thorough review of systems, laboratory testing is recommended and typically includes a CBC, LFTs, renal function tests, and urinalysis [1, 2].

Treatment for a primary episode of cutaneous vasculitis is aimed at avoidance of suspected triggers (in our patient, elasomeran vaccine), as well as supportive treatment such as leg elevation, antihistamines, and NSAIDs. For mild refractory LCV, antineutrophilic agents such as colchicine and dapsone are considered first-line agents [3]. Severe refractory LCV requires more potent systemic immunosuppressive agents such as mycophenolate mofetil, azathioprine, and tapering systemic corticosteroids [1–3]. In cases of vasculitis with extensive systemic involvement, a high risk of end-organ damage or death requires therapy with a combination of high potency corticosteroids and cyclophosphamide [3].

## 4. Conclusion

Our case highlights LCV as a potential novel side effect of the elasomeran vaccination. The case of LCV in our patient was self-limiting, however, the potential for a more severe immunological reaction with systemic involvement should be considered a possibility, and patients should be counseled of the risks associated with receiving the second dose of the elasomeran vaccine. In the case of our patient with a history of ITP, a predisposition for an amplified immunological response is considered as a potential confounding variable. Further research is required to isolate specific compounds within the vaccination associated with a high risk for an immunological reaction.

## Data Availability

The data that support the findings of this study are available and will be provided upon reasonable request.
